# Strengthening crisis resilience in German primary care by using quality indicators: findings of a process evaluation in the RESILARE project

**DOI:** 10.1186/s13690-024-01400-7

**Published:** 2024-10-08

**Authors:** Regina Poß-Doering, Jan Koetsenruijter, Nicola Alexandra Litke, Aline Weis, Martina Köppen, Stephanie Kümmel, Joachim Szecsenyi, Michel Wensing

**Affiliations:** 1 Department of General Practice and Health Services Research, University Hospital Heidelberg, University Heidelberg, Im Neuenheimer Feld 130.3, 69120 Heidelberg, Germany; 2 Section for Translational Medical Ethics, National Center for Tumor Diseases (NCT) Heidelberg, Heidelberg, Germany; 3 aQua Institute, Göttingen, Germany

**Keywords:** Crisis resilience, Climate change, Quality indicators, Primary care

## Abstract

**Background:**

In recent years, health systems worldwide have been confronted with several crises such as natural disasters or the COVID-19 pandemic, that affected lives and health of many people. In light of waves of infections and heat, climate change is considered to be the biggest health threat of the 21st century. Strengthening individual and organizational crisis resilience in healthcare settings thus becomes a crucial factor in maintaining care quality and protecting vulnerable patients during such crises. The RESILARE project therefore aimed to develop and evaluate quality indicators that support primary care practices in preparing for and adapting to crisis-related challenges.

**Methods:**

In a three-phased process, indicator development was based on systematic literature research and qualitative data, a two-stage expert panel process, and pilot testing in a maximum of *n* = 35 ambulatory practices during an outreach visit. Practice-individual indicator-related status and benchmarking information were provided via feedback reports to complete the audit and feedback program. A mixed-methods process evaluation used semistructured interviews with participating General practitioners and nonphysician health professionals to explore support and challenges for the implementation of the derived set of quality indicators. Two online surveys were conducted to evaluate all indicators and the two-part feedback report. Qualitative data were analyzed inductively using a thematic analysis approach. Survey data were analyzed descriptively.

**Results:**

A total of *n* = 32 indicators covered four domains: (1) individual resilience, (2) crisis prevention, (3) organizational resilience, and (4) climate resilience. *N* = 34 practices participated in the piloting and the process evaluation. Participants generally attributed a high relevance to the domains, and considered the indicator set suitable for implementation into existing quality management systems. Planning and implementation of measures that strengthen crisis resilience in practices were triggered or intensified by piloting the indicators and by the two-part feedback report. The identified challenges involved the volume of indicators and practice-individual implementation of renewable energy sources on rented premises. Participants expressed their desire for peer exchange regarding proven concepts for crisis resilience.

**Supplementary Information:**

The online version contains supplementary material available at 10.1186/s13690-024-01400-7.

**Table:** 

Text box 1. Contributions to the literature
• Maintaining structured action is crucial for ensuring care quality in crisis situations, particularly for vulnerable population groups.
• Applying quality indicators can support the identification of preventative measures and strengthen crisis resilience in primary care.
• Both medical and therapeutic care can benefit from blueprints for structured action and handling of stressful situations regarding individual, as well as organizational resilience.
• Sustainability thinking, crisis prevention and climate resilience need greater consideration in public health policy so adequate care can be provided to the public in crisis situations.
• A systemic resilience should be addressed through a strategy regarding potential disruptions.

## Background

Changes in climate and environment have dramatic impacts on population health worldwide [1–3]. Due to rising temperatures, extreme weather phenomena and further environmental changes, healthcare systems are facing new challenges such as disease outbreaks and heat waves. The majority of patients at risk for new types of infectious diseases and heat-related health problems are expected to be treated in the ambulatory sector. The teams in primary care practices play a central role in prevention strategies since they are often the first and only health contact points for the risk group of older people living alone [4]. By the end of the century, the frequency of heatwaves in Germany is expected to triple, and their duration is expected to increase by 25% [5]. This is projected to lead to a 2.4-fold increase in heat-related health problems, such as coronary heart disease [6, 7]. Health threats resulting from germs in drinking water or food and infectious diseases such as dengue fever are also expected in Germany in the future [8] or were already dramatically noticeable during the COVID-19 pandemic, posing new challenges for healthcare. Preparing primary care physicians and their teams for the challenges posed by crisis situations such as the increasing number of heat-related health problems, is therefore highly relevant for ensuring the quality of care for vulnerable patient groups during crises [9].

In German general practices, audit and feedback on the basis of quality indicators are used as improvement strategies with the aim of initiating standardized care processes and ensuring care quality [10]. Quality indicators can be defined as quantitative measures that provide information about effectiveness, safety and/or people-centeredness of care [11]. Quality indicators that address crisis situations in ambulatory practices are developed and evaluated in the RESILARE project (RESILARE - Building resilience of primary care practices by developing and evaluating quality indicators) to induce a standardized preparative strategy that strengthens handling of and coping with crisis situations and individual and organisational resilience. Moreover, RESILARE aims to provide an impetus for identifying starting points for reducing the ecological footprint of the ambulatory healthcare sector itself and contributing to respective overall efforts [12, 13].

The RESILARE project had three phases to derive a set of quality indicators, and prioritize, evaluate and pilot the indicators in general practices using an audit and feedback program. The present study describes findings of the accompanying process evaluation which aimed to explore relevance and perceived applicability of the piloted quality indicators, perception of the audit and feedback program and management of crisis situations from care provider perspective with regard to strengthening crisis resilience in primary care practices.

## Methods

### Design and context

**Fig. 1:**
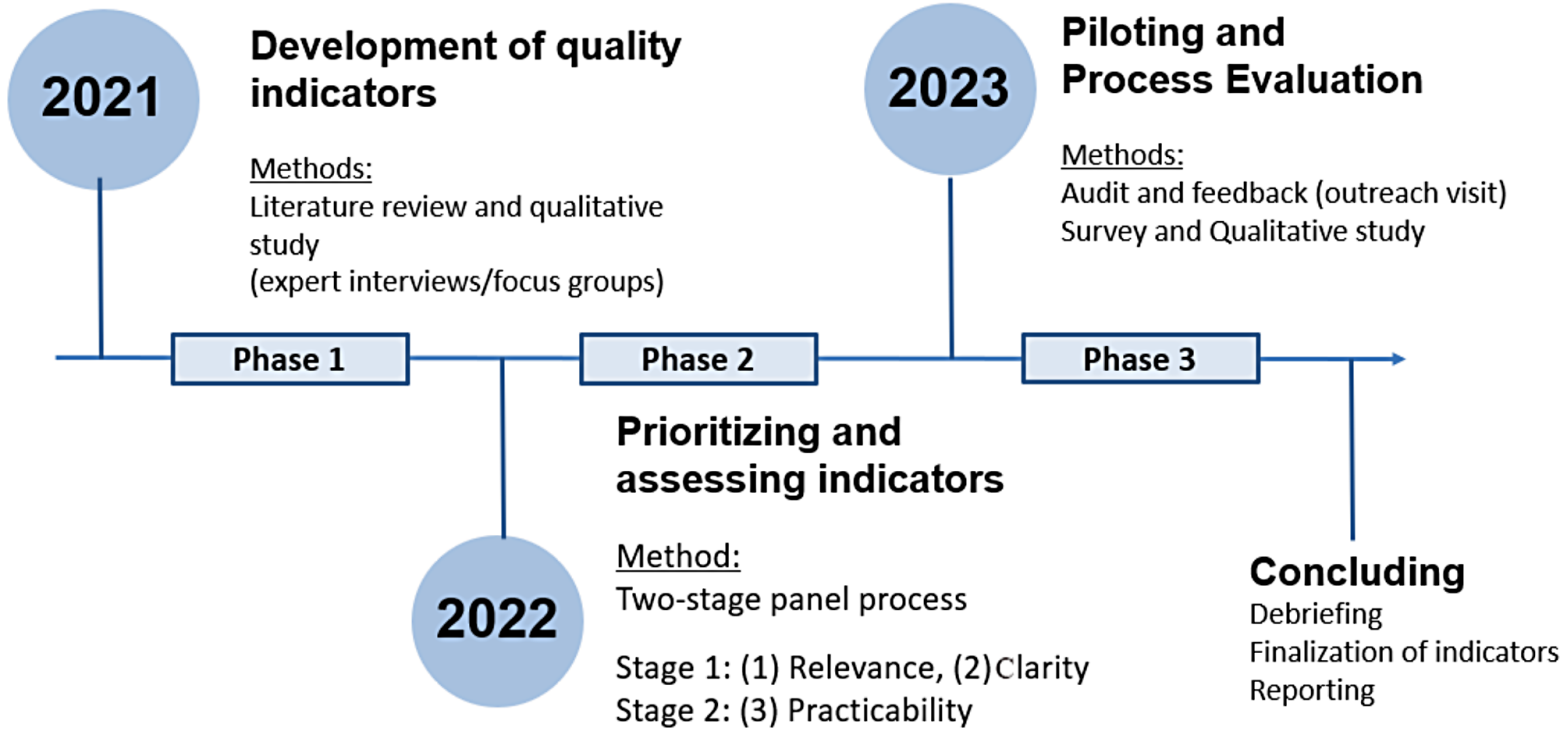
The RELIEF study phases (2021–2023) and corresponding applied methods

The RESILARE project used three study phases: (1) derivation of quality indicators via literature search and qualitative primary data collection, (2) prioritization and evaluation of derived indicators, and (3) piloting the indicators with associated feedback in general practices during an outreach visit accompanied by a process evaluation. In the first phase, potential crises and strategies were identified [12, 13]. Following the RUMBA model (Relevant, Understandable, Measurable, Behaviorable, Achievable) [14], *n* = 42 potential indicators in 4 domains were derived. During the second phase, indicators were assessed for relevance, clarity, plausibility, and practicability in a modified two-phased RAND/UCLA [15] panel approach with 15 experts, followed by prioritization and finalization of the indicator set. Subsequently, *n* = 32 indicators with 47 items were piloted in the third phase. Figure 1 provides an overview of the study phases and applied methods.

Process evaluations can be used to understand the functioning of an intervention by investigating the uptake of intervention components, mechanisms of impact and contextual factors [16]. As part of the third project phase, the process evaluation in RESILARE aimed to assess applicability and relevance of the piloted indicators and to identify determining organizational and individual factors impacting a potential implementation into quality management. The process evaluation was designed as a mixed-methods observational study which was linked to a program of audit and feedback in primary care practices. In a convergent-parallel design, a quantitative survey study and a qualitative study were conducted using two study-specific online questionnaires, semistructured interviews, and a feedback form for outreach visitors.

To pilot the quality indicators (Supplementary Table 1), an auditing outreach visit was planned to be conducted with each participating practice either on-site or via an online video platform, and optionally during regular quality management certification. Such an accreditation is a method for assessing and benchmarking the performance of general practice care across a broad range of clinical and organizational domains [17]. To assess the status quo, experienced outreach visitors (physicians or medical assistant professionals) were asked to present general information on crisis resilience and go through the RESILARE indicators with participants. Feedback reports were to be issued related to (a) practice-individual assessment regarding the RESILARE indicators and (b) benchmarking.

### Study sample

Recruitment was conducted by the aQua Institute for Applied Quality Improvement and Research in Health Care, Göttingen, Germany, as responsible project consortium leader in RESILARE. A purposive sample of general practices were invited to participate in the pilot. The sampling strategy considered practices of different sizes and forms of organization (single, group, or shared practices and professional associations), and in different implementation stages of quality management (re-certification, certification, starter) from all over Germany. Practices were invited via phone, e-mail, newsletter, website, or personally.

All practices were asked to sign an agreement with the aQua Institute covering participation in an outreach visit (on-site or via online platform) and piloting of the indicators, the process evaluation, and remuneration after completion. Participation was intended for one General practitioner and one nonphysician health professional – comparable to medical assistants (MAs) in USA [18] - per practice. After agreeing to piloting the quality indicators, physicians named one designated MA in their practice after obtaining respective consent. All members of a participating practice team could join in the outreach visit. Only participants who were able to give written and signed consent, older than 18 years and in sufficient command of German were included in the process evaluation.

### Data collection and measures

Within two weeks of outreach visit and piloting the indicator set, a practice-individual feedback report was provided online by the aQua Institute via a link to their proprietary platform VISOTOOL^®^ [19] to describe performance status quo regarding the RESILARE indicators. For the process evaluation, all participating General practitioners (GPs) and Medical Assistants (MA) were invited via e-mail to rate all piloted indicators in an online survey approximately two weeks after receiving the first part of the feedback report. Survey data collection used two online study-specific questionnaires developed by the study team responsible for the process evaluation at the Department of General Practice and Health Services Research, University Hospital Heidelberg, Germany.

The first questionnaire covered the complete set of developed indicators and facilitated a scaled rating of each domain as well as of all indicators with regard to relevance and acceptability for everyday practice. Also included were items concerning difficult situations at the workplace, cost and relevance of sustainable ways of working, open questions on coping with current crises, as well as socio-demographic characteristics. Items in the second questionnaire referred to the feedback reports and already derived improvement measures. Table 1 further describes the included items.

**Table 1:** Items included in survey 1 and 2 in 2023 and their scaling

Survey items	Rating	Number of items	Scale
All domains All indicators	Relevance Applicability Clarity	4 32 32	1–9 1 = not relevant at all 9 = very relevant
Difficult situations at work	Handling/Coping	16	1–7 1 = not applicable at all 7 = fully applies
General perception	Expenditure and objective consistency Cost and relevance of sustainability	3 2	1–5 1 = very high 5 = very low
Current crises	Coping	3	Open questions
Feedback report	Comprehensiveness, transparency, stimulus General comments Derived improvement measures	7 2 1	1–9 1 = does not apply at all 9 = fully applies Open question

Participants received a personalized link to each survey questionnaire via e-mail. A reminder regarding the first questionnaire was sent via e-mail six weeks after the last outreach visit. All outreach visitors were asked to rate perception of acceptance and sustained implementation of the indicators with 4 items on a scale from 1 to 10 (1 = negative − 10 = positive) and could provide free text on 2 open questions on challenging and enabling factors during the visit.

All participants were then contacted via postal mail to invite them to participate in a telephone interview. Invitations were supplemented with two copies of a consent form and written information on data protection, process evaluation, and study aims. Participants were asked to return one signed copy of the written consent for participation in the process evalaution. Subsequently, they were contacted via telephone by the study team to clarify potential questions and agree on date and time for the interview. If participants were not reached by phone after several attempts, e-mail were sent with interview date suggestions. Qualitative data collection was based on a semistructured interview guide with pre-defined open questions covering outreach visit, feedback report, measures applied based on impulses provided by the piloting, currently perceived crises and motivation for participation in the RESILARE project. The interview guide (Additional file 2, Supplementary Table 2) was developed by the study team responsible for the process evaluation at the Department of General Practice and Health Services Research, University Hospital Heidelberg, Germany. Questions for physicians and MAs were the same, though exact wording and order were individually adapted during interviews. A verbal reminder regarding the survey questionnaire was provided after each interview. To minimize time expenditure for participants and pursue data economy, participant characteristics were collected via survey 1 only.

All telephone interviews were audio recorded, pseudonymized and transcribed verbatim. Handwritten notes on relevant aspects were taken during all interviews. No interview was interrupted, repeated or cancelled. Transcripts were generated using the AI-based software noScribe Version 0.3 [20]. All transcripts were proofread (first author and student support staff) and amended where applicable. The qualitative data collection was conducted by the first author, a health services researcher and implementation scientist with extensive experience in qualitative research. Survey data collection was led by JK, an experienced health services researcher and data scientist. Visitor feedback collecting was coordinated by MK, an experienced quality management accreditation expert. After conclusion of all visits, the second part of the feedback report provided benchmarking.

### Data analysis

To explore perceptions referring to relevance and acceptability of the domains and indicators, the audit and feedback program, and coping with current crises, IBM SPSS Statistics Version 29.0.0.0 was used for analysis of survey and sociodemographic data. Data were checked for plausibility by two experienced study team members and data cleaning was applied where applicable. Subsequently, data were analyzed descriptively (JK) to summarize central tendency (mean), variability (range; standard deviation), and frequency (count; percentage). Only answered survey items were included, missing values were marked by a specific code. Data visualization was done in Microsoft Excel 2019.

A two-step approach based on Thematic Analysis [21] was applied to analyze the qualitative data regarding perception of indicators and domains, audit and feedback program, derived improvement measures, motivation for participation, and coping with current crises. First, a data subset of 5 transcripts of interviews with GPs and MAs each were coded inductively by two female health services researchers with profound experience in qualitative research. Initial codes were then checked against the interview guide themes and matched with them and final coding was applied to all remaining transcripts. Divergent codings were discussed in regular meetings to achieve a wide consensus and high intercoder congruity. Methodological strategies such as seeking for similarities and differences across and within accounts were applied to ensure representation of different perspectives, trustworthiness of analysis, and transparency of findings [22]. To organize and manage the qualitative data, MAXQDA 2022 Plus (Release 22.7.0) software (Verbi GmbH) was used. Quantitative and qualitative data were analyzed separately first and then brought together to complement each other.

## Results

Findings derived from both quantitative and qualitative process evaluation data are presented focusing on perceived relevance and applicability of the RESILARE indicators and covered domains, perception of outreach visit and feedback report, and management of crisis situations. Provided quotes from the qualitative data are presented with an indication of alias, transcript number and position (Pos.), and have been translated with due diligence.

### Participant characteristics

A total of *n* = 57 GPs were initially interested in participation. *N* = 34 GPs and *n* = 34 MAs from *n* = 35 practices of varied sizes and located throughout Germany could be recruited to participate in the piloting and the process evaluation and signed a written consent. All other interested GPs declined participation due to workload. One GP cancelled participation shortly before the scheduled visit and past completion of recruitment due to exceptionally high workload and staff shortage. One practice participated with two sites but the same GP and MA, and completed questionnaires and interviews once each. *N* = 40 participants completed the first and *n* = 35 the second questionnaire. Between April 25 and September 05, 2023, a total of *n* = 65 telephone interviews with GPs (*n* = 33) and MAs (*n* = 32) were conducted (mean duration 21 min; range 11–42 min). During data collection, participants were either at their workplace, commuting or at home. One GP elaborated on questions supported by the practice manager and no MA from this practice was interviewed due to insufficient language skills. Table 2 details the participant characteristics.

**Table 2:** Characteristics of the participants in the piloting and the process evaluation (*n* = 34 general practices in Germany)

Participants	*N*
General Practices Single practice n (%) group practice n (%) Shared/professional association n (%) urban/rural n (%)	34 12 (35) 15 (44) 7 (21) 16/18 (47.1/52.9)
Survey 1 Gender (m/f) Age mean (range; SD) Age range 40–49 years n (%) Professional experience years mean (min/max) Working hours/week mean (min/max) Directing work-related instructions n (%)	40 18/22 28.8 (26–68; 11.8) 14 (35) 24.4 (5/43) 41.5 (20/70) 31 (77)
Interviews Physicians n (m/f) Medical Assistants n (f)	65 33 (21/12) 32 (32)

Participants stated they felt motivated to participate in piloting the indicators because they wanted to broaden and strengthen current efforts regarding crisis resilience and sustainable healthcare provision by learning about further approaches. They also felt motivated by the combination with accreditation of the obligatory quality management. A small number of GPs mentioned that remuneration for participation was a decisive factor. Some MAs described that the GP had asked them to jointly participate because of their specific roles in the team or prior efforts and they felt motivated by that.

### Relevance and applicability of domains and RESILARE indicators

**Fig. 2:**
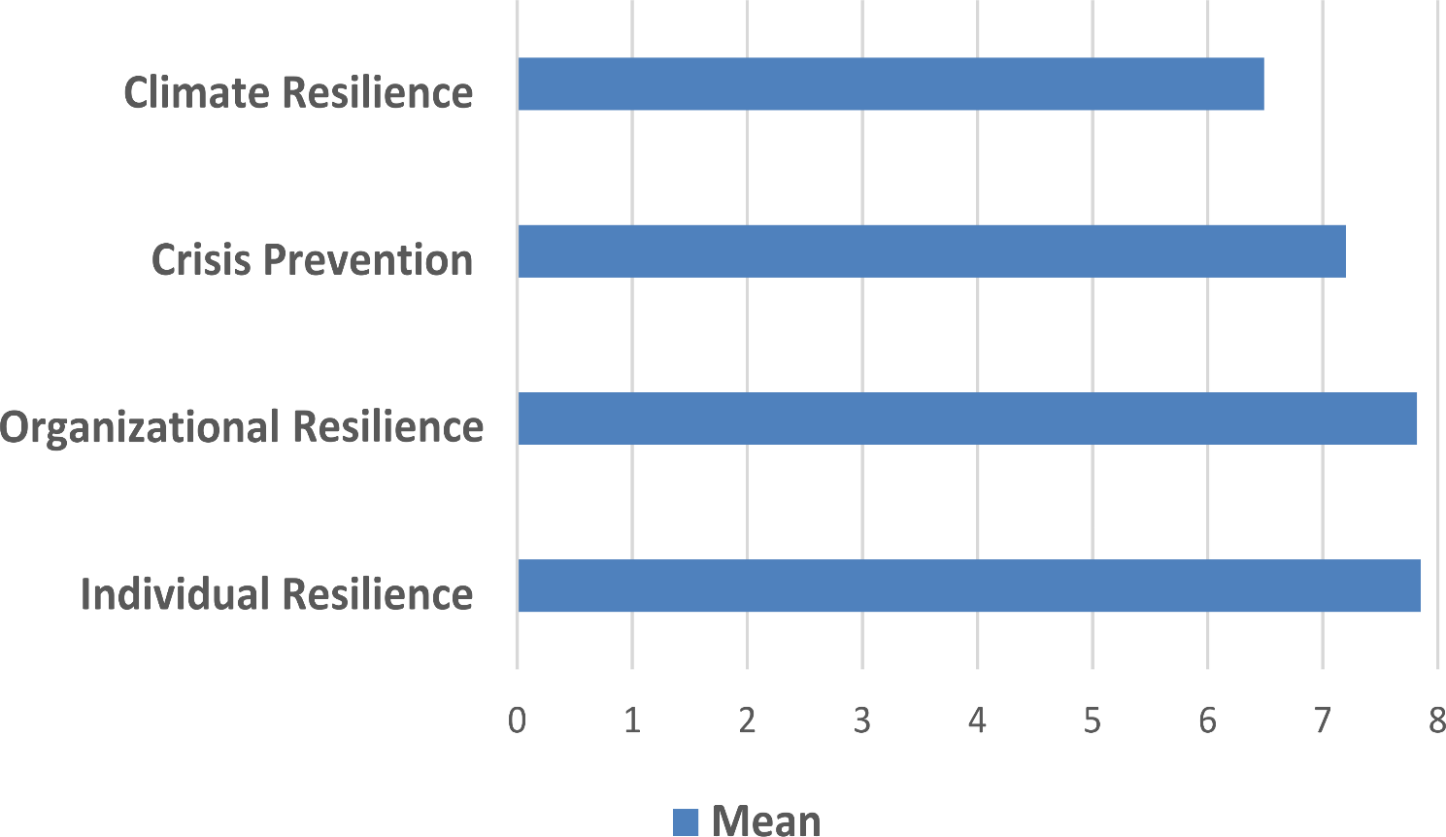
Relevance attributed by the participants (*n* = 40) in 2023 to the four addressed domains Scale from 1 to 9: 1 = not relevant at all − 9 = very relevant

**Fig. 3:**
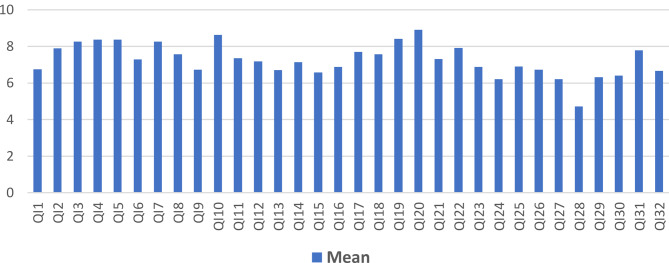
Relevance attributed to all RESILARE indicators by the participants (*n* = 37–40) in 2023 Scale from 1 to 9: 1 = not relevant at all − 9 = very relevant

Survey questionnaire 1 asked the participants to indicate relevance they attributed to the four domains in general and referring to each single indicator. On a scale from 1 to 9 (1 = not relevant at all − 9 = very relevant), the domains regarding *Individual resilience* (mean 7.85; SD 0.81) and *Organizational resilience* (mean 7.82; SD 0.91) were rated the highest, followed by Crisis *Prevention* (mean 7.2; SD 1.29) and *Climate Resilience* (mean 6.49 SD 2.01). All indicators were rated above 6 with the exception of the one referring to measuring the ecological footprint of a practice (mean 4.72; SD 2.64)). Figures 2 and 3 visualize these findings.

On a scale from 1 to 5, relevance of sustainable ways of working in ambulatory care was rated neutral (mean 2.28; SD 1.01). The overall objective consistency of the indicators was rated similarly (mean 2.26; SD 0.55). Suggestions for specification of indicators in free text fields mainly referred to providing examples to facilitate transparency regarding content and intention of an indicator at first glance, for instance referring to safety concepts, measures of strengthening individual resilience, potential risks and the formation of local networks. Some participants indicated their individual and skeptical views on feasibility of some indicators, particularly referring to political or economic context, competencies and capacities in primary care, and ecological footprint of practices.

The qualitative data largely matched survey findings regarding perceived applicability and relevance of the indicators and the covered domains. Participants stated they considered the indicators to be “very well grounded and absolutely relevant for all practices” (GP08, Pos. 21) and feasible for implementation in mandatory quality management programs. Two GPs and two MAs mentioned that standardized indicators might be difficult to implement since practices differed individually and thus some indicators might not correspond with daily practice. Some participants recommended a gradual implementation to provide sufficient space and time for practices to design and prepare corresponding measures and processes. Generally, participants considered it important for practices to reflect on the domains, particularly on climate change resilience and sustainable care provision, and emphasized a need to individually consider what could be changed in the practice regarding climate action and be made aware of potential starting points.

So, it’s very important, and I thought it was truly good to pause for a moment and of course the environmental aspect is very important, in many things your hands are tied, but in some things, I think, […] if you just knew. (GP04, Pos. 25).

Some GPs and MAs mentioned that they had been considering and engaging regularly in aspects covered by the indicators for some time, particularly in connection to quality management in the practice, but also in their private lives. Few participants self-critically reflected to neglect these important topics sometimes during daily routines. While considering three of the domains relevant, one GP stated that resilience to climate change would not play a role in the particular region where the practice was located and that sustainable care was inept. One MA noted that “Environment and climate are a bit difficult and a double-edged sword in medicine” (MA33, Pos.13) since so many single-use items were present. Regarding implementation and use of the indicators, critical views referred mainly to existing hygiene regulations as well as uncertainty about relevance for primary care in general or for the particular practice.

At first, I thought it was a bit excessive, I have to say, to add this to basic medical care, but over time, to be honest, the deeper you delve into this subject, the more sense it makes to think about it. (MA06, Pos. 23).

### Outreach visit and feedback report

Outreach visits were conducted via video calls (*n* = 22), on-site (*n* = 10), and over telephone due to technical difficulties (*n* = 2). The quality management experts who conducted the visits indicated their positive perceptions regarding resonance in the practice teams and general acceptance of the indicators (9.4 and 8.6; scale 1–10). They also considered implementation of key messages into daily practice very probable (9.2) and attributed a high influence of the indicators on future decisions in the practices (9.1). In the survey, overall expenditures for preparation, attendance and follow-up of the outreach visit were classified with a mean of 2.45 (scale 1–5; SD 0.81). Regarding the feedback reports, 57% of participants indicated full comprehensibility, 77% confirmed clarity. 80% welcomed the benchmarking information and indicated that they had initiated improvement measures based on their visit and report. Free text fields were used to detail measures implemented after the visit and feedback on incident and crisis management, team building, conservation of energy, patient information material and negotiations with landlords.

Interview participants generally described the outreach visit as a very targeted, informative and useful tool for piloting the indicators. The time invested was mostly considered adequate and corresponding to perceived benefits of the assessed status quo and impetus for reflection, planning and implementing necessary measures. Some practices opted to combine the piloting with quality management accreditation to be mindful of resources. Participants reported that this made a separate contemplation of the piloting somewhat difficult for them and the visitor.

We not only took part in the RESILARE study during the visitation, but it was also part of our [quality management] certification, so it was a bit difficult for both, the visitor and us to separate the two, because some of the content simply overlapped […], I think we understood the impact of the questions very well. (GP22, Pos. 5).

GPs and MAs explained they thought it was a good idea to pursue sustainability thinking not only during an ongoing crisis, but also focus on it in a preparatory way during the outreach visit. It was mentioned that the visit and feedback report provided confirmation for being on the right path regarding potential improvement measures. Most participants described their expectation to receive additional impulses and learn about best practices via the feedback report. While some GPs expected to benefit from the benchmarking, others mentioned they would appreciate specific suggestions, instructions for implementation of adequate measures and an exchange of ideas with other practices. Few GPs had no expectations regarding the feedback report. Some GPs and MAs had not accessed the first part of the online feedback report at the time they were interviewed, thus the second part which included the benchmarking was mailed by postal service to avoid an information gap.

Based on insights gained through outreach visits and feedback reports, GPs and MAs alike reported that they had started to compile to-do lists for their planning of improvement measures and discussed them during their regular team meetings. It was considered important to include the whole team in corresponding efforts. Some participants described that the visit had taken place shortly before a vacation period and planning for improvement measures had yet to be initiated. Measures already planned or implemented referred to active transportation for house calls and getting to work, team building and communication, appreciative interaction and individualized working hours, sustainable and climate resilient care provision, supply management and reduction of single-use materials, safety concepts and heat protection plans, patient information, emergency planning and potential power outages, digitalization of health services, energy resources, waste sorting, incident handling, adaptation of practice hours, solar shading and energy-saving air-conditioning during summer months, ventilation during winter months, energy-saving light sources, and data storage. Some GPs planned to implement new measures after moving to different premises.

I’m currently building a new practice, and I’m already able to implement a lot of the energy aspects and optimize CO_2_ savings. And the fact that we are actually addressing the issue of bicycles more, our role model function, and that we’re also making sure that we motivate patients to come to the practice on foot or by bike, that the issue of sustainability is more important than parking spaces. (GP16, Pos. 31).

### Managing critical situations

Survey 1 asked participants’ self-perception regarding their ability to manage critical situations. The minimum rating across items was 1, the maximum was 7 (1 = not applicable at all − 7 = fully applicable), and the mean score ranged from of 5.61 to 6.2. A mean score of 6 and above was registered for a total of 6 items. Table 3 details these items, mean scores and standard deviations.

**Table 3:** Participants’ self-perception of handling crisis situations (*n* = 38–40)

Item	*N*	Min	Max	Mean	SD
At work, …					
I address challenges by contemplating potential actions.	40	3	7	6.2	0.97
if I get too excited, I can calm myself so I can go on.	39	3	7	6.13	1.08
I generally look at difficult situations from different perspectives.	39	1	7	6.03	1.22
when there are difficult tasks, I focus on my goal and won’t be deterred.	39	3	7	6	1
difficulties provide opportunity to use my skills.	39	3	7	6	1.17
I thoroughly consider my actions before tackling a problem.	38	3	7	6	1.09

Regarding current crises, participants in the interview study felt resilience was a daily essential. They outlined approaches to coping with medication shortages. Time-consuming communication efforts were described, both with patients and pharmacies when a specific medication was not available and prescriptions had to be re-issued. For most practices, this was perceived as a daily occurrence, resource-intensive for the team and frustrating for patients. Most GPs and MAs stated to routinely communicate with pharmacies in the region to assess availabilities on a daily basis. Some practices phoned pharmacies before patients went there with a prescription, others received daily availability updates from pharmacies or information was passed on through patients. One GP perceived a positive side effect when fewer antibiotics were available since less prescribing contributed to reducing the risk of antimicrobial resistance. Another GP felt that medication shortages changed perceptions regarding sustainability aspects when people realized they could manage with less. One practice asked patients to deposit unused nonexpired medication with them to be able to pass them on to patients in need. GPs were also aware that medication shortages could result in non-guideline-compliant therapy, potential harm for patients, and otherwise avoidable hospitalization.

The sheer number of patients we look after means we have an enormous administrative burden because about every second prescription comes back corrected by the pharmacy, or we have to make phone calls to pharmacies. We really experienced an almost pandemic level of bacterial tonsilitis, and almost no penicillin was left in the entire district. […]. So, it is difficult to provide very good care in line with guidelines if the medication is simply unavailable. And to explain this to the patients, who are usually truly nice anyway, but the frustration increases, you have to be honest. (GP21, Pos. 31).

Only a few participants felt that they were not confronted with crisis situations related to energy supply and costs, patients affected by war and migration, and inflation while most GPs and MAs felt affected daily and could detail their handling of related crisis situations. It was mentioned that crisis situations were addressed and discussed calmly with patients when they were brought up or when an impression arose that counseling was indicated. GPs voiced concerns regarding the economic well-being of their practice and teams and stated that it was important to be aware of employer responsibilities and the protective role of a GP practice. It was also noted that with higher cost of living, some patients might not be able to afford necessary medication. One GP mentioned that MAs often learned more about traumatizing crisis situations from patients when they changed a wound dressing than GPs did during a consultation. As crisis response, such incidents were then discussed in team meetings to share the burden and develop coping strategies.

Well, it is demonstrated to you every day how important resilience is. Because it is so omnipresent, you’re confronted with these crisis issues every day […] I have to be prepared if things do not work out. […] And that we actually pass this on to the patients. (GP16, Pos. 39).

Regarding inflation and rise of costs, participants shared to apply cost-cutting strategies such as increasing the use of renewable energy sources, reducing overall energy consumption for instance by switching off ultrasound and other devices, sustainable supply management and the use of electromobility for house calls. Participants also contemplated the bigger picture beyond their own practices, patients and teams and voiced that regarding current crises they noticed a feeling of helplessness, insecurity and anxiety about future developments, overstraining, depression, and loss of a carefree world around them and for themselves.

Ultimately, we have to assess what this means for us, for example the blasting of a dam, the water does not get here directly, but it has an impact, […] we are talking about electric cars and CO_2,_ and what happens there in one day - I exaggerate - is sometimes what happens in Germany in a year, but we are talking about environmental disasters and similar things, and what is happening there is a sheer environmental disaster, and it all has to do with us, also in the long term, it does not pass us by. (GP20, Pos. 23).

## Discussion

In a novel approach, the RESILARE intervention piloted 32 quality indicators related to crisis resilience in primary care. GPs and MAs who participated in the corresponding process evaluation considered the RESILARE indicators relevant and applicable for primary care practices and feasible for implementation in mandatory quality management. Individual and organizational resilience as well as crisis prevention were seen as more urgent aspects than climate resilience. All participating practices described using audit and feedback for the piloting as appropriate and time invested as beneficial regarding status quo, reflection, planning and implementing necessary measures. When the visit was combined with quality management accreditation, it was perceived to be somewhat difficult to contemplate the RESILARE indicators separately.

In a recent report, the German Expert Council for the Assessment of Developments in the Healthcare System focused on crisis resilience and stated that the system’s self-perception of being well-organized and prepared for unexpected developments was deceptive [23]. The expert council assumed that nature and frequency of crisis-related challenges cannot be predicted with certainty and thus recommended to follow an “all hazards approach” [24] and to strengthen the “health in all policies” [25] principle. The council also recommended that a resilience strategy regarding potential disruptions should aim for systemic resilience, and cover a preparatory phase, timely detection, impact and coping, and recreation and learning. The preparatory phase outside of times of crisis was seen as crucial for taking preventive measures in good time and anticipating, detecting and managing potential crises at an early stage [23]. The set of quality indicators developed and piloted in the RESILARE project covers the postulated systemic “all hazards approach” and precisely focuses on what can be done in preparatory phases before potential disruptions might turn into a crisis situation for practices.

Researchers and policy-makers seek to develop and use systematic ways of measuring and benchmarking quality of care which is systematically reported as part of health system performance reports in many countries. Measuring quality of care provides the basis for quality assurance and improvement strategies. In particular, accreditation and certification, audit and feedback, public reporting and pay for quality are considered to rely on availability of robust information about the quality of provided care to determine the extent to which new regulations or quality improvement interventions work and actually facilitate improvements [11]. The combination of audit and feedback is a widely used strategy in interventions aiming to improve professional practice and can be a component of multifaceted quality improvement interventions to prompt healthcare professionals to modify practice where feedback provides indications [26, 27]. In the RESILARE intervention, outreach visits were used as audits since they are considered to be a credible instrument in continuous medical education [26, 28], can improve the care delivered to patients [29] and might be applied when changes are considered to be difficult to achieve [30]. Effects can be expected to be small to moderate and are likely to be greater when baseline adherence to a recommended practice is low and when feedback is delivered in a more intensive way [27]. In this present study, feedback reports were provided via an online platform (part 1) and in a print version (part 2) to disseminate relevant information regarding practice-individual performance and benchmarking and point out improvement potential relating to crisis resilience, both on the organizational and on the individual level, which has been perceived as fundamental for responding to a crisis [12]. This prompted reflection and planning of measures to strengthen crisis resilience. It therefore seems reasonable to assume that a broad integration of the RESILARE indicators into quality management systems combined with intensive performance feedback could generate progress monitoring for individual practices on a regular basis, and potentially also build a robust information base regarding crisis resilience across ambulatory care.

It can be assumed that healthcare providing entities have an effect on care processes, which in turn will influence patient outcomes. It is also to be assumed that crisis resilience is crucial for maintaining quality and availability of care. Given the complexity of healthcare provision and the wide range of relevant aspects it entails, quality management systems often use a larger number of indicators to measure and monitor [11], which might make it difficult for practices to implement all at the same time to prepare for crisis situations. Findings of this process evaluation indicate that participating practices shared this concern and therefore suggested a gradual introduction into quality management. Since the RESILARE indicator set covers four self-contained domains, such a step-by-step approach could be followed by considering and implementing one domain at a time.

The domain of climate resilience was considered less relevant for primary care practices than the other three domains. However, human behavior has changed climate and environment over the last decades in such a profound way that climate change will shape people’s future behaviors as serious health-related threats are posed to current and future generations [31]. Health-related behaviors could have a significant positive impact on climate change, for instance regarding actions targeting mitigation and adaptation [32]. High-impact mitigation actions include the use of active transportation such as riding a bike or walking, instead of using individual cars [31]. Several participating practices reported using active transportation for house calls and to get to work, which was seen as an economic necessity. Largely confirming prior findings [13], their focus was rather on recycling of materials, reducing waste, or saving energy by turning off appliances when not in use: environmentally friendly behaviors with low mitigation potential that serve the comfort of believing in sufficiency of current individual efforts [31]. Critical leverage points do include rethinking consumption and waste, exploring alternative visions of good quality of life, and promoting education and learning, all of which would benefit health [33, 34]. Health professionals are asked to be powerful advocates for tackling climate change for the sake of health and recognize this crisis as a global health emergency [33]. To facilitate awareness and a proactive culture in primary care practices, climate change-related adaptation strategies should be perceived as part of healthcare provider roles rather than as add-ons to already high workloads [35]. This could contribute to realizing unused potential, shaping the role model function regarding a significant contribution to climate change-related behavior, recognizing the relevance of this domain more clearly, and conveying it to patients in a more targeted way. Besides implementing relevant indicators into quality management, addressing these aspects in peer networks, quality circles and continued education could promote exchange and a more comprehensive awareness of the necessity of crisis resilience in primary care.

### Strengths and limitations

Findings of this process evalaution contribute to the growing body of knowledge on the relevance of resilience in the context of primary care. In a novel approach, the piloted RESILARE indicators address dimensions of individual and organizational resilience, crisis prevention, and climate resilience to strengthen the resilience of primary care practices, which goes far beyond aspects of coping with ongoing crises. Piloting the RESILARE indicators, subsequent feedback on status quo and benchmarking prompted reflection and planning of measures regarding crisis resilience. However, it must be assumed that practices did not share the same baseline and thus, perceived effects on practice level are likely to be individual and potentially small. Reporting of this process evaluation is based on recommendations in the Consolidated Criteria for Reporting Qualitative Studies (COREQ) checklist [36].

Some limitations have to be considered. It is possible that the sample might underlie a sampling bias toward practices with pronounced interest in the topic of resilience and the impact of systemic disturbances in the health sector. Participants might have delved into the topic more intensively prior to piloting the indicators which might have resulted in a divergent baseline. The RESILARE indicators were developed for use in ambulatory practices and piloted in GP practices only. It is possible that other medical specialists and their teams could contribute different insights than the observed sample.

## Conclusion

The RESILARE indicators might contribute to the systematic strengthening of crisis resilience in primary care practices and to maintaining high care quality during disruptions. Implementation in certified quality management should heed identified potential challenges to avoid overstraining of practices.

## Electronic supplementary material

Below is the link to the electronic supplementary material.

**Supplementary Table 1**: Aspects addressed by RESILARE indicators

**Supplementary Material 2**: Interview guide - RESILARE process evaluation (translated)

## Data Availability

The data that support the findings of this study are not openly available due to reasons of sensitivity and are available from the corresponding author upon reasonable request. Data are located in controlled access data storage at the Department of General Practice and Health Services Research, University Hospital Heidelberg, Germany.

## Abbreviations

COREQ: Consolidated Criteria for Reporting Qualitative Studies
GP; GPs: General practitioner; General practitioners
MA; MAs: Medical Assistant; Medical Assistants
Pos: Transcript position
