# Supplementary material for: Strengthening crisis resilience in German primary care by using quality indicators: findings of a process evaluation in the RESILARE project
Source: Arch Public Health. 2024 Oct 8;82:177. doi: 10.1186/s13690-024-01400-7 (PMC11460109; doi:10.1186/s13690-024-01400-7)
Supplement: Supplementary file 1 — Supplementary Table 1: Aspects addressed by RESILARE indicators [file 13690_2024_1400_MOESM1_ESM.pdf]

Supplementary Table 1: Aspects addressed by RESILARE indicators

Quality Indicator	Aspect addressed
**Domain 1 - Individual resilience**	
**1**	Implementation of measures regarding self-empowerment, mindfulness, continued education
**2**	Team building
**3**	Team meetings, reflection of disturbances
**4**	Team meetings in acute crisis situation, information flow
**5**	Prioritizing and redistribution of tasks in crisis situation
**6**	Protective concept for team, de-escalation training, dealing with aggravated patients, information flow
**7**	Keeping up team satisfaction, appreciative, constructive feedback
**8**	Complaints management, constructive error management
**Domain 2 – Crisis Prevention**	
**9**	Educational measures regarding resilience and sustainability
**10**	Emergency response training
**11**	Risk identification
**12**	Initiating measures in crisis situation
**13**	Individual action plans
**14**	Dissemination of content of action plans
**15**	Insurance check
**Domain 3 - Organizational resilience (practice level)**	
**16**	Communication channels with patients
**17**	Regional networking
**18**	Quality circles
**19**	External contacts
**20**	Data back-up and storing
**21**	Alternatives in case of IT failure
**22**	Supply chain management and stock keeping
**Domain 4 - Climate resilience**	
**23**	Sustainable, climate-friendly work processes, dissemination of related concept
**24**	Patient information related to health-related climate aspects
**25**	Climate and health in counselling
**26**	Climate-related illnesses, related training
**27**	Individualized heat protection plan, dissemination of plan
**28**	Calculation of ecological footprint
**29**	Renewable energy sources
**30**	Active and sustainable mobility
**31**	Reasonable purchasing and use of materials
**32**	Prescribing of sustainable medication, rational use of medication
