# Supplementary material for: Strengthening crisis resilience in German primary care by using quality indicators: findings of a process evaluation in the RESILARE project
Source: Arch Public Health. 2024 Oct 8;82:177. doi: 10.1186/s13690-024-01400-7 (PMC11460109; doi:10.1186/s13690-024-01400-7)
Supplement: Supplementary file 2 — Supplementary Material 2: Interview guide - RESILARE process evaluation (translated) [file 13690_2024_1400_MOESM2_ESM.pdf]

Supplementary Table 2: Interview guide - RESILARE process evaluation (translated)

**Topics**	**Covered aspects**
**Part 1**	
Cost and benefits of piloting	Format of outreach visit (online; on-site)<br>Aspects addressed in depth
Relevance of domains and quality indicators	What was missing regarding crisis resilience?<br>Perspectives on potential nation-wide use of indicators
**Part 2**	
Insights gained via visit and piloting of indicators	regarding adaptation of processes<br>ecologically sustainable aspects of care provision
Measures initiated to strengthen crisis resilience	Who initiated?<br>What has changed?<br>Further planning for climate-friendly care provision?<br>Individual behaviors
**Part 3**	
Feedback report	Expected impetus<br>Practice-individual, benchmarking
Current crises	Energy supply, inflation, war<br>Impact on practice and team<br>Mitigating measures<br>Challenges
**Part 4**	
Closing	Motivation for participation in RESILARE<br>What kind of support is desired?<br>Final comments regarding indicators and study

:
